# The impact of residual vessel fractional flow reserve on clinical outcomes: The FAST residual study^☆^

**DOI:** 10.1016/j.ijcha.2026.102022

**Published:** 2026-10-06

**Authors:** Karol Sadowski, Jari A. van der Eijk, Mariusz Tomaniak, Annemieke C. Ziedses des Plantes, Wijnand K. den Dekker, Roberto Diletti, Rutger-Jan Nuis, Jeroen Wilschut, Isabella Kardys, Nicolas M. Van Mieghem, Joost Daemen

**Affiliations:** a Department of (Interventional) Cardiology, Thoraxcenter, Erasmus University Medical Center, Rotterdam, the Netherlands.; b First Department of Cardiology, Medical University of Warsaw, Warsaw, Poland

**Keywords:** Coronary artery disease, Angiography-based fractional flow reserve, Percutaneous coronary intervention

## Abstract

**Backgrounds:**

Vessel fractional flow reserve (vFFR) allows angiography-based physiological lesion evaluation and virtual percutaneous coronary intervention (PCI) simulation, estimating post-PCI vFFR (residual vFFR). While residual vFFR has been validated against invasive post-PCI fractional flow reserve (FFR), its clinical significance remains unclear. To assess the impact of residual vFFR on long-term clinical outcomes.

**Methods:**

FAST Residual is a post hoc analysis of the FFR SEARCH study including 279 patients with (un)stable angina or non-ST-segment elevation myocardial infarction undergoing angiographically successful PCI. Residual vFFR was calculated based on two pre-procedural angiographic views. The primary endpoint was target vessel failure (TVF) at 5 years, a composite of cardiac death, target vessel myocardial infarction (TVMI) and target vessel revascularization (TVR).

**Results:**

Mean age was 64 ± 11 years, 68.5% were men, 23.7% had diabetes, and 43.7% presented with stable angina. Median residual vFFR was 0.92 [IQR 0.87–0.95] and post-PCI FFR was 0.90 [0.85–0.94] (*r* = 0.509, *p* < 0.001). The optimal cutoff value of residual vFFR to predict 5-year TVF was <0.90. TVF occurred in 24 (23.3%) patients in the residual vFFR <0.90 group, compared with 16 (9.1%) in the residual vFFR ≥0.90 group (adjusted HR 2.66, 95% CI 1.41–5.04; *p* = 0.003). This difference was driven by higher rates of TVR [16 (15.5%) vs. 12 (6.8%); adjusted HR 2.33, 95% CI 1.10–4.94; *p* = 0.027].

**Conclusions:**

The concept of virtual stenting is a promising tool for predicting clinical outcomes. For the first time, this post hoc analysis showed that patients with residual vFFR <0.90 experienced significantly higher rates of TVF at 5 years.

## Graphical abstract

HR: Hazard Ratio, NSTEMI: Non-ST Elevation Myocardial Infarction, PCI: Percutaneous Coronary Intervention, Res vFFR: Residual Vessel Fractional Flow Reserve, TVF: Target Vessel Failure, TVMI: target vessel myocardial infarction, TVR: Target Vessel Revascularization, vFFR: vessel Fractional Flow Reserve.

**Figure:**
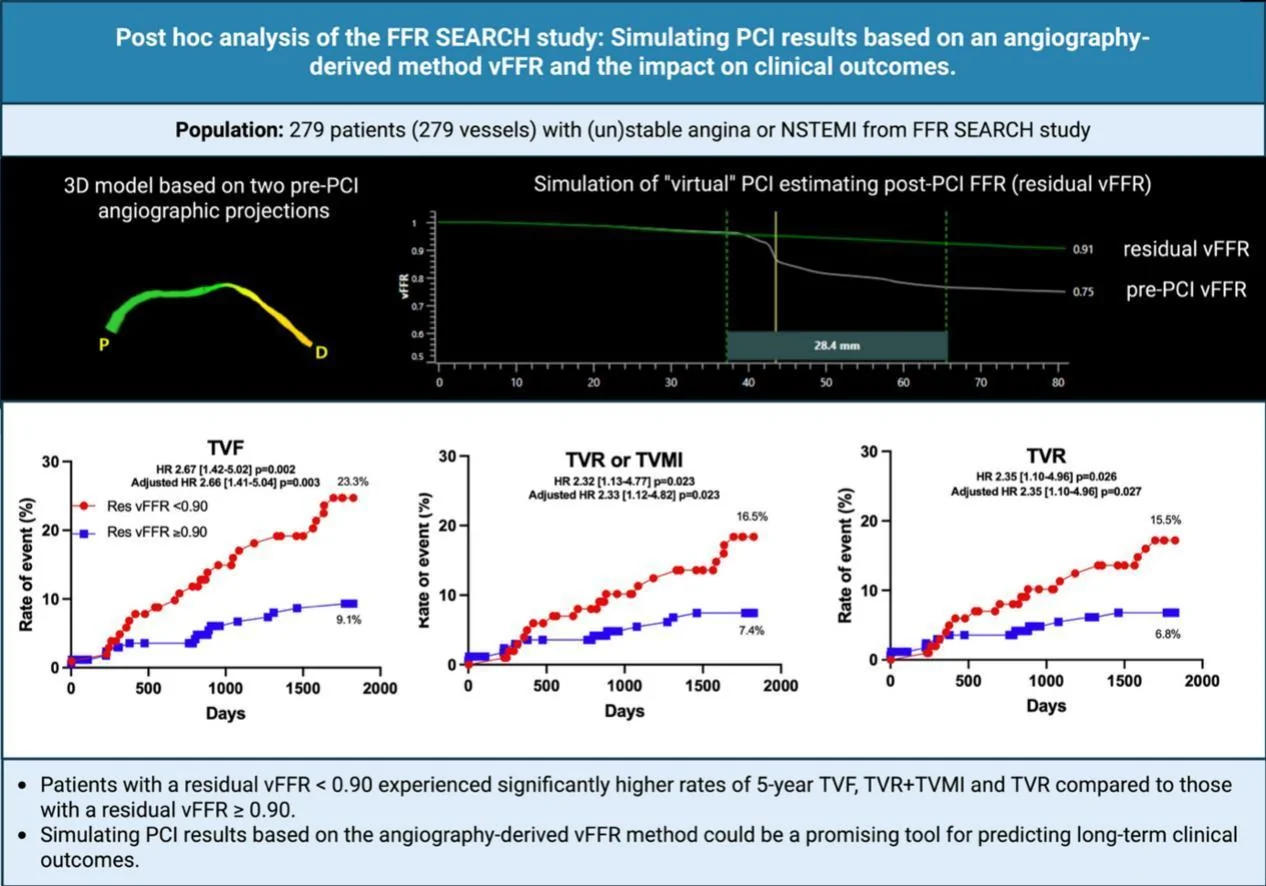

## 1 Introduction

Functional lesion assessment after angiographically successful percutaneous coronary intervention (PCI) has shown significant prognostic value [1], [2]. Specifically, lower post-PCI fractional flow reserve (FFR) have been associated with worse clinical outcomes [3], [4], [5], [6], [7], [8].

Vessel fractional flow reserve (vFFR) has recently been validated as an accurate, less-invasive alternative to conventional pressure-wire-based FFR for functional assessment of intermediate coronary artery lesions by using a three-dimensional (3D) reconstruction of coronary angiography images in both pre- and post-PCI settings [9], [10], [11]. Recent advancements allow for the simulation of ‘virtual’ PCI, estimating post-PCI FFR (residual vFFR) from pre-procedural angiographic images. Residual vFFR is calculated by virtually resolving the stenosis and the subsequent pressure drop across the lesion of interest, assuming optimal stent placement. Whereas the diagnostic accuracy of residual vFFR has been validated against post-PCI FFR, its clinical relevance remains elusive [12]. The aim of this study was to evaluate the impact of residual vFFR values on clinical outcomes.

## 2 Methods

The FAST Residual study is a post-hoc analysis of the FFR SEARCH study and aims to determine the impact of residual vFFR on clinical outcomes. The detailed study design and protocol of the FFR SEARCH registry have been previously described [13], [14]. In brief, FFR SEARCH was a prospective registry, obtaining FFR measurements after angiographically successful PCI in 1000 consecutive patients between March 2016 and May 2017 at the Erasmus University Medical Center, Rotterdam, the Netherlands. Inclusion criteria for the present substudy included age ≥ 18 years, presentation with either stable or unstable angina or non-ST elevation myocardial infarction, at least one significant stenosis in one of the epicardial coronary arteries (diameter stenosis of ≥70% by quantitative coronary angiography (QCA) or hemodynamically significant stenosis defined as FFR <0.80) and presence of two orthogonal angiographic projections allowing successful vFFR computation. Patients presenting with ST-segment elevation myocardial infarction (STEMI), left main lesions or bypass grafts to the target vessel were excluded. Our study adhered to the principles of the Declaration of Helsinki and received approval from the local medical ethical committee. All participants provided written informed consent.

### 2.1 FFR measurements

Post-PCI FFR was measured using a dedicated microcatheter (Navvus RXi™, ACIST Medical Systems, Eden Prairie, MN, USA). FFR was defined as the mean distal coronary artery pressure divided by mean aortic pressure during maximum hyperemia induced by continuous intravenous infusion of adenosine at a rate of 140 μg kg^−1^ ·min^−1^ through an antecubital vein [14]. Post-PCI FFR was assessed at 2 cm distal from the most distal stent edge [14]. Subsequently, two standard monoplane angiographic views (≥30^°^ apart, preferably orthogonal) were obtained after a bolus of 200 μg of nitroglycerine. For each patient, the position of the pressure wire was recorded. Aortic root pressure was continuously recorded, and the pressure measurement registered before the FFR measurement was utilized as input in the vFFR software [9].

### 2.2 Pre-PCI vFFR calculation and residual vFFR analysis

Residual vFFR analyses were performed offline by a certified analyst (KS) using dedicated software (CAAS workstation 8.5.5 software, Pie Medical Imaging, Maastricht, The Netherlands). The methodology of pre-PCI vFFR calculation has been previously reported [9], [10], [11]. Residual vFFR was calculated from pre-PCI angiograms at the pre-specified location of the FFR pressure sensor in post-PCI angiograms. The vessel contour delineation was carried out semi-automatically from the ostium. While the alignment of the cardiac cycle between the two angiograms was performed automatically using ECG triggering, manual frame selection was allowed in case of excessive overlap during the automatically defined optimal end diastolic frame [9]. The percentage diameter stenosis, minimal lumen diameter, reference lumen diameter, minimal lumen area, and lesion length were derived from the same 3D model used to estimate the (residual) vFFR [9]. No manual adjustments were made for lesion location, estimated stent length and stent placement.

### 2.3 Study endpoints

The primary endpoint was target vessel failure (TVF) at 5 years, defined as a composite of cardiovascular death, target vessel myocardial infarction (TVMI), and target vessel revascularization (TVR). Secondary endpoints included TVR or TVMI, TVMI, TVR, cardiovascular death and stent thrombosis (ST). The target vessel was defined as the vessel in which residual vFFR was computed and post-PCI FFR was assessed. Cardiac death was defined as any death in which a cardiac cause could not be excluded [15]. TVR was defined as a re-intervention driven by any lesion in the same epicardial vessel. Myocardial infarction (MI) was defined according to the fourth universal definition of MI [16]. TVMI was defined as a re-MI driven by any lesion in the same epicardial vessel. ST was defined according to the Academic Research Consortium 2 definitions [15]. Event adjudication was performed by 2 independent cardiologists unaware of the final physiological assessment.

### 2.4 Data collection

All baseline and procedural data were retrieved from the hospital's electronic medical record system and recorded in a specialized electronic database. Clinical follow-up was obtained for each patient from electronic medical records of the hospital, general practitioner, and municipal civil records databases. In addition, all patients were contacted personally by letter or telephone. The treating physician or general practitioner was asked for additional information to complete follow-up data if necessary.

### 2.5 Statistical analysis

Continuous variables were expressed as mean ± standard deviation for normally distributed variables or as median with interquartile range for non-normally distributed variables. The Shapiro-Wilk test was used to assess the normality of distributions. Continuous variables were compared using the Student's *t*-test for normally distributed variables and the Mann-Whitney *U* test for non-normally distributed variables. Categorical variables were compared using Pearson's chi-square test or Fisher's exact test, as appropriate. Correlation analyses were conducted using Spearman's rank correlation coefficient.

In patients with multiple interrogated study vessels, the vessel with lowest residual vFFR was used for the vessel-level analysis and was considered the target vessel [1]. Cumulative event rates were derived from the Kaplan-Meier function. Censoring was performed at the time last contact, or after 5 years of follow-up (whichever occurred first). The optimal residual vFFR and post-PCI FFR cutoff value for the primary endpoint (TVF) were obtained based on the maximum log-rank statistic. The patients were divided based on the optimal residual vFFR cutoff value to predict 5-year TVF. Sensitivity analyses were also performed using alternative residual vFFR thresholds of 0.88 and 0.92 in addition to the data-derived threshold of 0.90. The association between residual vFFR and clinical endpoints was analyzed using Cox proportional hazard regression analysis. First, univariable analysis was performed. Subsequently, predictors showing significant differences between groups were included in multivariable models adjusted for a set of potential confounders chosen based on clinical relevance and a previous study. The following variables were considered in the multivariable models: sex, hypertension, dyslipidemia, and diabetes, depending on the number of events [13], [17]. Residual vFFR was additionally analyzed as a continuous variable using Cox proportional hazards regression, with HRs expressed per 0.01 decrease. To assess the robustness of the association between residual vFFR <0.90 and 5-year TVF, additional Cox regression models were constructed incorporating prior MI and prior PCI; LAD location and stent length; and LAD location, ACC/AHA B2/C lesion complexity, and stent length. All associations are presented as hazard ratios (HRs) with 95% confidence intervals (CIs). Statistical analyses were performed using the SPSS statistical package version 24 (IBM, Armonk, North Castle, NY, USA) and Prism GraphPad 10 (GraphPad Software, Inc., CA). A 2-sided *p* < 0.05 was considered statistically significant.

## 3 Results

### 3.1 Study population

Pre-PCI vFFR and residual vFFR measurements were successfully performed in 279 vessels. The FFR SEARCH study enrolled 1000 patients in which post-PCI FFR was successfully measured in 959 patients. Within this subset, 345 patients were excluded due to ST segment elevation myocardial infarct (STEMI) as the presenting aetiology (375 vessels), left main disease (19 vessels), and treatment of graft vessels (10 vessels). Following angiographic assessment, 179 patients (272 vessels) were excluded due to the lack of 2 adequate pre- procedural orthogonal views of the study vessel allowing (residual) vFFR computation. vFFR analyses was not possible for an additional 159 patients (186 vessels) due to overlap, insufficient angiographic quality or contrast opacification, table movement, foreshortening, or poor contrast filling. The flowchart of this study is presented in Fig. 1.

**Fig. 1:**
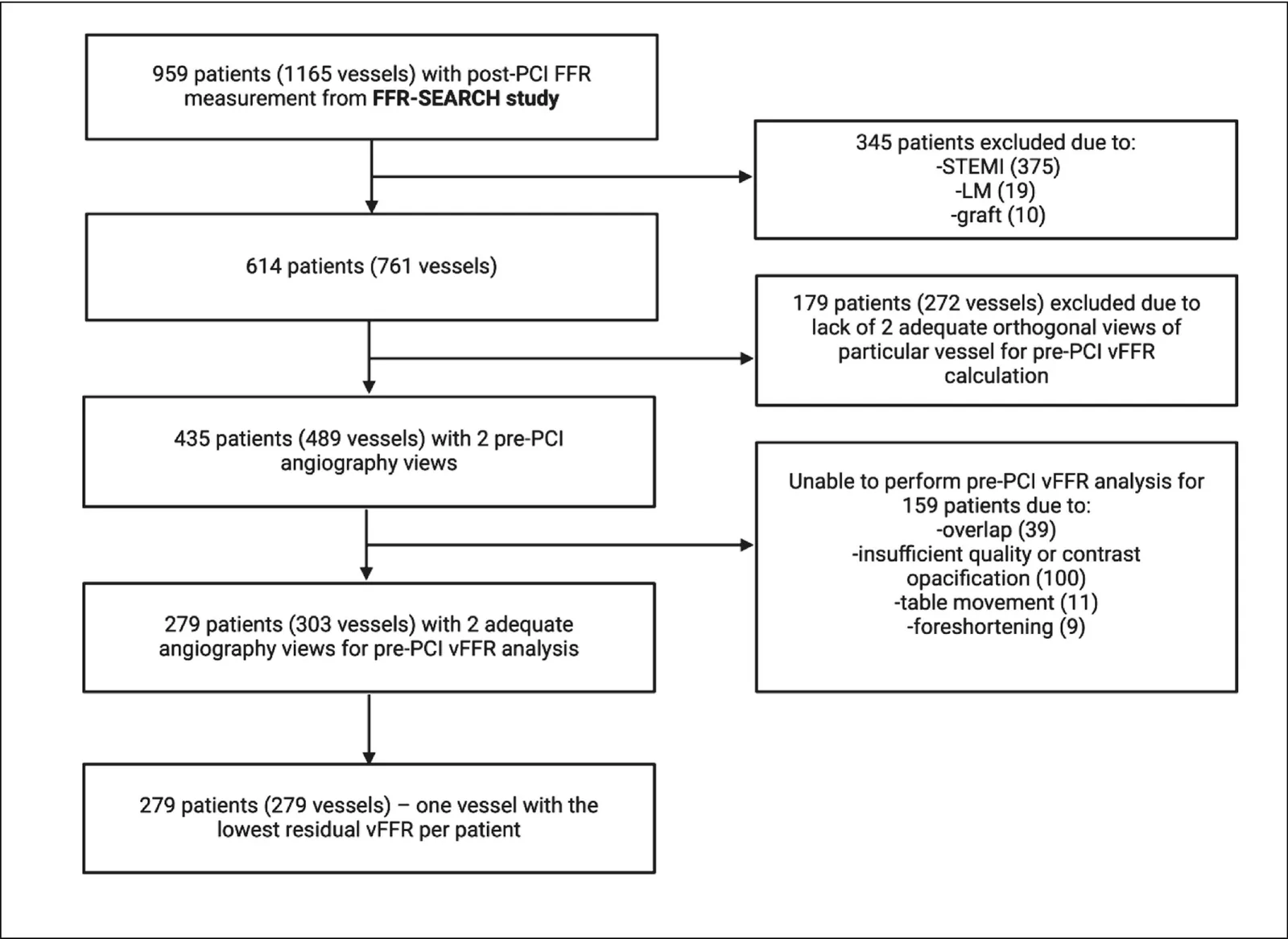
Study flow chart. Values in brackets indicate the number of excluded vessels. FFR, fractional flow reserve; vFFR, vessel fractional flow reserve; STEMI, ST elevation myocardial infarction; LM, left main; PCI, percutaneous coronary intervention.

### 3.2 Baseline characteristics

Baseline clinical characteristics are presented in Table 1, while procedural characteristics are summarized in Table 2. In brief, the mean age was 64 ± 11 years, 23.7% of patients had diabetes, 62.7% of the coronary lesions were classified as type B2/C. Median stent length was 22 mm [IQR, 15–35] and median post-stenting 3D minimum luminal diameter was 2.49 mm [IQR, 2.18–2.81].

**Table 1:** Baseline Clinical Characteristics.

	All patients (*n* = 279)	Residual vFFR <0.90 (*n* = 103)	Residual vFFR ≥0.90 (*n* = 176)	*p*-value
Age, y	63.97 (±10.79)	65.25 (±11.05)	63.22 (±10.60)	0.13
Male sex (n)	191 (68.5%)	74 (71.8%)	117 (66.5%)	0.35
Cardiovascular risk factors, n (%)				
Hypertension	169 (60.8%)	62 (60.2%)	107 (61.1%)	0.88
Hypercholesterolemia	153 (55.4%)	64 (62.7%)	89 (51.1%)	0.06
Diabetes	66 (23.7%)	26 (25.2%)	40 (22.7%)	0.63
Current smoker	131 (47.0%)	46 (44.7%)	85 (48.3%)	0.56
Prior stroke	24 (8.6%)	10 (9.7%)	14 (8.0%)	0.61
Peripheral arterial disease	23 (8.2%)	14 (13.6%)	9 (5.1%)	0.013
Comorbidity, n (%)				
Prior myocardial infarction	67 (24.0%)	34 (33.0%)	33 (18.8%)	0.007
Prior PCI	83 (29.7%)	40 (38.8%)	43 (24.4%)	0.011
Prior CABG	11 (3.9%)	6 (5.8%)	5 (2.8%)	0.22
Creatinine, μmol/L, median (IQR)	82.00 (72.00–98.25)	82.00 (73.00–99.00)	82.00 (71.00–98.00)	0.60
Hb level, mmol/L, median (IQR)	9.00 (8.00–9.00)	9.00 (8.00–9.00)	9.00 (8.00–9.00)	0.14
Presentation, n (%)				
Stable angina	122 (43.7%)	47 (45.6%)	75 (42.6%)	0.62
Unstable angina/NSTEMI	157 (56.3%)	56 (54.4%)	101 (57.4%)	0.62

Vessel-based analysis. Data are reported as mean ± SD or median and IQR. CABG, coronary artery bypass grafting; Hb, Haemoglobin; NSTEMI, Non-ST Elevation Myocardial Infarction; PCI, percutaneous coronary intervention.

**Table 2:** Procedural Characteristics.

	All vessels with residual vFFR (n = 279)	Residual vFFR < 0.90 (n = 103)	Residual vFFR ≥0.90 (n = 176)	p-value
Lesion type by American College of Cardiology (ACC)/American Heart Associations (AHA), n (%)				
A	40 (14.3%)	5 (4.9%)	35 (19.9%)	<0.001
B1	64 (22.9%)	16 (15.5%)	48 (27.3%)	0.024
B2/C	175 (62.7%)	82 (79.6%)	93 (52.8%)	<0.001
Bifurcation	38 (13.6%)	17 (16.5%)	21 (11.9%)	0.28
Calcified	117 (41.9%)	46 (44.7%)	71 (40.3%)	0.48
Thrombus	23 (8.2%)	11 (10.7%)	12 (6.8%)	0.26
Measured vessel, n (%)				
RCA	60 (21.5%)	17 (16.5%)	43 (24.4%)	0.12
LAD	171 (61.3%)	83 (80.6%)	88 (50.0%)	<0.001
LCx	44 (15.8%)	2 (1.9%)	42 (23.9%)	<0.001
IM	4 (1.4%)	1 (1.0%)	3 (1.7%)	0.62
QCA measurements; median (IQR)				
Stenosis pre, %, mean ± SD	54.91 (±16.43)	52.36 (±15.97)	56.38 (±16.56)	0.043
Stenosis post, %, mean ± SD	3.56 (±13.82)	4.33 (±15.27)	1.84 (±13.39)	0.52
MLD pre, mm	1.14 (0.81–1.43)	1.12 (0.80–1.42)	1.16 (0.83–1.45)	0.81
MLD post, mm	2.49 (2.18–2.81)	2.32 (2.11–2.66)	2.60 (2.20–2.94)	0.002
Stent length, mm	22.00 (15.00–35.00)	30.00 (20.00–44.00)	21.00 (15.00–28.00)	<0.001
Stent diameter, mm	3.00 (2.75–3.50)	3.00 (2.75–3.25)	3.00 (2.78–3.50)	0.003
No. of stents, n	1.00 (1.00–1.00)	1.00 (1.00–2.00)	1.00 (1.00–1.00)	<0.001
Predilatation, n (%)	187 (67.0%)	74 (71.8%)	113 (64.2%)	0.19
Postdilatation, n (%)	189 (68.0%)	72 (70.6%)	117 (66.5%)	0.48
Use of IVUS	36 (12.9%)	15 (14.6%)	21 (12.0%)	0.54
Post-PCI FFR	0.90 (0.85–0.94)	0.86 (0.81–0.90)	0.94 (0.86–0.96)	<0.001
Pre-PCI vFFR	0.76 (0.63–0.84)	0.71 (0.58–0.79)	0.79 (0.68–0.88)	<0.001
Residual vFFR	0.92 (0.87–0.95)	0.86 (0.81–0.88)	0.92 (0.92–0.96)	<0.001

Data are reported as mean ± SD or median and IQR. CTO, chronic total occlusion; FFR, fractional flow reserve; IM, intermediate artery; IQR, interquartile range; IVUS, intravascular ultrasound; LAD, left anterior descending artery; LCx, left circumflex artery; MLD, minimum luminal diameter; PCI, percutaneous coronary intervention; QCA, quantitative coronary angiography; RCA, right coronary artery; and vFFR, vessel fractional flow reserve.

The median calculated pre-PCI vFFR was 0.76 [IQR, 0.63–0.84] whereas the median residual vFFR was 0.92 [IQR, 0.87–0.95] and the median post-PCI FFR was 0.90 [IQR, 0.85–0.94] (*r* = 0.509, *p* < 0.001) (Fig. 2). The correlation between residual vFFR and post-PCI FFR was moderate (*r* = 0.507, *p* < 0.001) (Fig. 3). The mean difference between residual vFFR and post-PCI FFR was 0.015, with 95% limits of agreement (−0.116 to 0.146). The optimal cutoff value to predict 5-year TVF was <0.90 for residual vFFR (*n* = 103/279, 36.9%) and < 0.89 for post-PCI FFR (*n* = 120/279, 43.0%) (Supplementary Figure. 1).

**Fig. 2:**
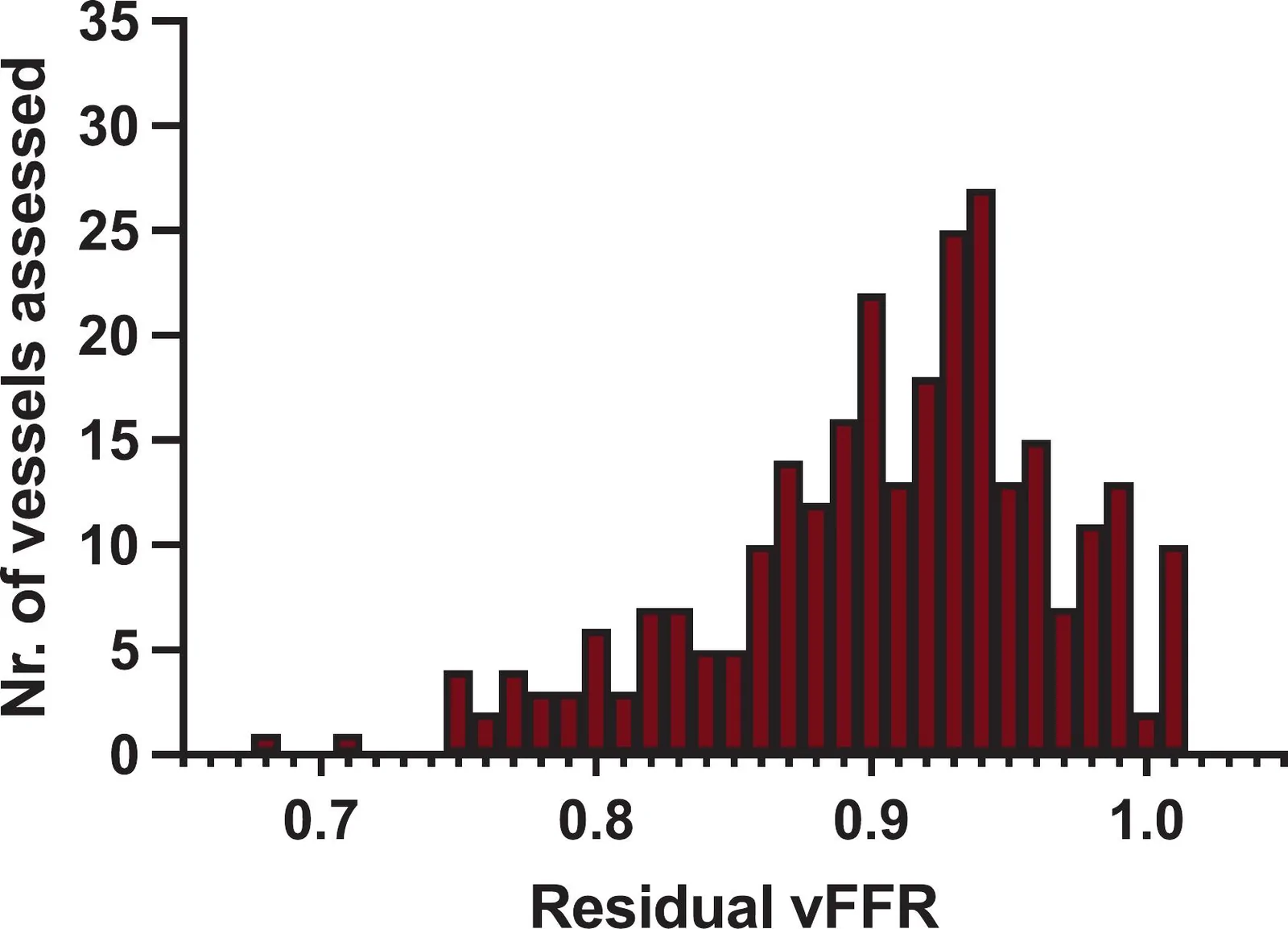
Vessels distribution per 0.01 residual vFFR increment. vFFR, vessel fractional flow reserve.

**Fig. 3:**
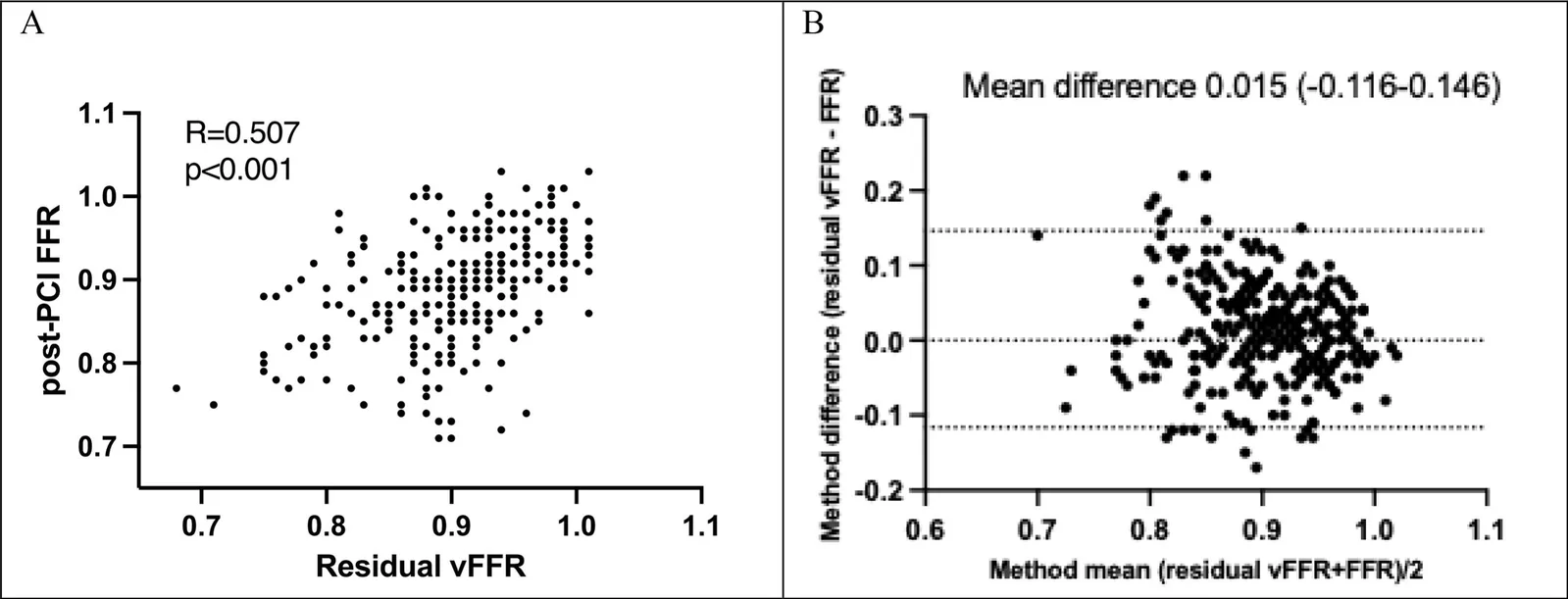
Association between post-PCI FFR and residual vFFR. (**A**) Scatter plot showing the correlation between post-PCI FFR and residual vFFR (*R* = 0.507, *p* < 0.001). (**B**) Bland–Altman plot demonstrating the mean difference between residual vFFR and post-PCI FFR (0.015), with 95% limits of agreement (−0.116 to 0.146). FFR, fractional flow reserve; vFFR, vessel fractional flow reserve; PCI, percutaneous coronary intervention.

Patients with residual vFFR <0.90 were more likely to have a history of prior PCI (38.8% vs. 24.4%; *p* = 0.011) and prior MI (33.0% vs. 18.8%, respectively; *p* = 0.007) compared with patients with a residual vFFR ≥0.90. Vessels with residual vFFR <0.90 more frequently included type B2/C lesions (79.6% vs. 52.8%, respectively; *p* < 0.001) and were more often located in the left anterior descending artery (LAD) (80.6% vs. 50.0%, respectively; *P* < 0.001) (Table 1).

Vessels with residual vFFR <0.90 were characterized by smaller pre-procedural diameter stenosis (52.4 ± 16.0 vs. 56.4 ± 16.6), *p* = 0.043 respectively) and smaller post-procedure minimum luminal diameter by 3D QCA (2.32 [IQR 2.11–2.66] vs. 2.60 [IQR 2.20–2.94], *p* = 0.002, respectively). Vessels with residual vFFR <0.90 received longer stents, with a median length of 30 mm (IQR, 20–44) vs. 21 mm [IQR 15–28], *p* < 0.001, and smaller stent diameters of 3.00 mm [IQR, 2.75–3.25] vs. 3.00 mm [IQR 2.78–3.50], *p* = 0.003, respectively. Vessels with residual vFFR <0.90 received more stents (1.00, IQR 1.00–2.00 vs. 1.00, IQR 1.00–1.00, *p* < 0.001). Finally, there was no difference in either the pre- or post-dilatation rates between groups (71.8% vs. 64.2%, respectively; *p* = 0.19) and (70.6% vs. 66.5%, respectively; *p* = 0.48), respectively (Table 2).

A sensitivity analyses comparing excluded patients as compared topatients included in the final residual vFFR analysis revealed largely similar baseline characteristics between both cohort (Supplementary Table 3). However, at a vessel levelseveral differences emerged. Analyzable vessels were more frequently located in the LAD (57.4% vs. 43.4%; *p* < 0.001) and less frequently in the LCx (19.1% vs. 26.6%; *p* = 0.017), had angiographically less severe lesions, larger MLD resulting in fewer and shorter stents. (Supplementary Table 4). Of interest, 5-year vessel-related outcomes were comparable between between patients in– and excluded in the final residual vFFR analyses (Supplementary Table 5).

### 3.3 Clinical outcomes

At 5 years, both univariable and adjusted analyses revealed a higher rate of TVF in the residual vFFR <0.90 group compared with the residual vFFR ≥0.90 group [24 (23.3%) vs. 16 (9.1%), respectively; HR 2.67, 95% CI 1.42–5.02; *p* = 0.002; adjusted HR 2.66, 95% CI 1.41–5.04; *p* = 0.003]. Cardiac death occurred in 8 (7.8%) and 6 (3.4%) patients, respectively (HR 2.29, 95% CI 0.80–6.63; *p* = 0.12). The rate of TVR or TVMI was higher in patients with residual vFFR <0.90 [17 (16.5%) vs. 13 (7.4%); HR 2.32, 95% CI 1.13–4.77; *p* = 0.023; adjusted HR 2.33, 95% CI 1.12–4.82; *p* = 0.023]. Similarly, TVR occurred in 16 (15.5%) vs. 12 (6.8%) patients (HR 2.35, 95% CI 1.10–4.96; *p* = 0.026; adjusted HR 2.33, 95% CI 1.10–4.94; *p* = 0.027). There were no significant differences in TVMI [4 (3.9%) vs. 7 (4.0%); HR 0.96, 95% CI 0.28–3.29; *p* = 0.95] or ST [1 (1.0%) vs. 3 (1.7%); HR 0.56, 95% CI 0.06–5.37; *p* = 0.61].The results of clinical outcomes for assessing the impact of residual vFFR are summarized in Table 3 and Fig. 4. When residual vFFR was analyzed as a continuous variable, each 0.01 decrease was associated with a higher risk of 5-year TVF (adjusted HR 1.06, 95% CI 1.01–1.11; *p* = 0.012), as well as TVR or TVMI (adjusted HR 1.06, 95% CI 1.01–1.11; *p* = 0.022) and TVR (adjusted HR 1.06, 95% CI 1.00–1.11; *p* = 0.037) (Table 4).

**Table 3:** Clinical Outcomes at 5-year Follow-Up.

	Residual vFFR <0.90	Residual vFFR ≥0.90	Univariate		Multivariate	
	N = 103	N = 176	HR (95% CI)	p-value	Adjusted HR⁎ (95% CI)	p-value
Cardiac death	8 (7.8%)	6 (3.4%)	2.29 (0.80–6.63)	0.12		
TVF	24 (23.3%)	16 (9.1%)	2.67 (1.42–5.02)	0.002	2.66 (1.41–5.04)	0.003
TVR or TVMI	17 (16.5%)	13 (7.4%)	2.32 (1.13–4.77)	0.023	2.33 (1.12–4.82)	0.023
TVR	16 (15.5%)	12 (6.8%)	2.35 (1.10–4.96)	0.026	2.33 (1.10–4.94)	0.027
TVMI	4 (3.9%)	7 (4.0%)	0.96 (0.28–3.29)	0.95		
Stent thrombosis	1 (1.0%)	3 (1.7%)	0.56 (0.06–5.37)	0.61		

HR, hazard ratio; TVMI, target vessel myocardial infarction; TVF, target vessel failure; TVR, target vessel revascularization.

⁎ Multivariable models were constructed only for outcomes with a statistically significant unadjusted association. TVF was adjusted for sex, hypertension, dyslipidemia, and diabetes; TVR or TVMI for sex, diabetes, and dyslipidemia; and TVR for sex and diabetes. For the remaining endpoints, adjustment was not performed because the number of events was too low to allow reliable multivariable modelling.

**Fig. 4:**
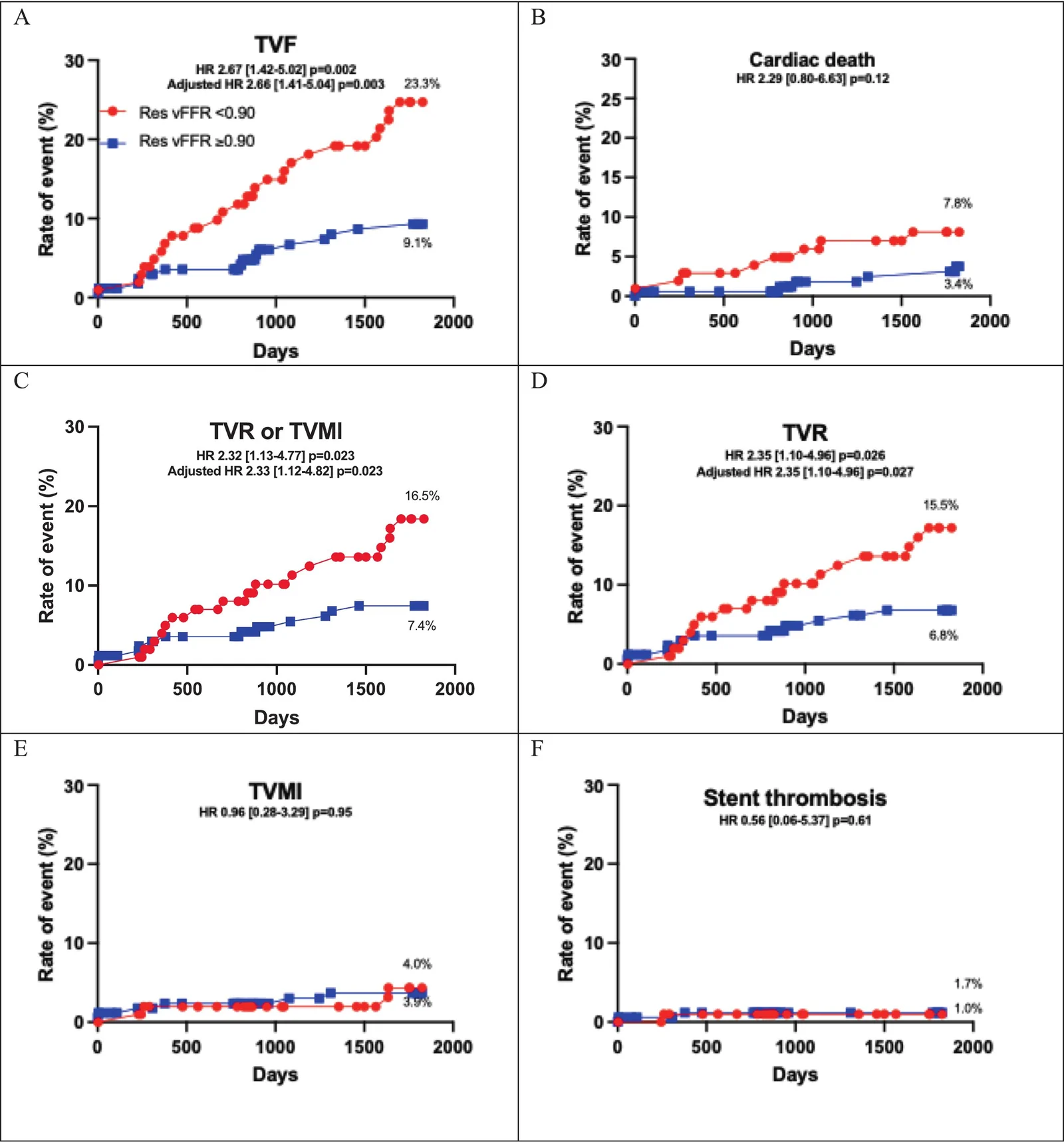
Kaplan-Meier curves based on residual vFFR for TVF (**A**), cardiac death (**B**), TVR or TVMI (**C**), TVR (**D**), TVMI (E) and stent thrombosis (F). The adjusted confounders were: for TVF – sex, hypertension, dyslipidemia, and diabetes; for TVR or TVMI – sex, diabetes, and dyslipidemia; and for TVR – sex and diabetes. HR, hazard ratio; TVF, target vessel failure; TVMI, target vessel myocardial infarction; TVR, target vessel revascularization; res, residual; vFFR, vessel fractional flow reserve.

**Table 4:** Association Between Residual vFFR as a Continuous Variable and 5-Year Clinical Outcomes.

Clinical outcome	Events, n (%)	Univariate HR (95% CI)	p-value	Adjusted HR⁎ (95% CI)	p-value
TVF	40 (14.3%)	1.060 (1.015–1.106)	0.008	1.058 (1.012–1.105)	0.012
Cardiac death	14 (5.0%)	1.033 (0.956–1.116)	0.411		
TVR or TVMI	30 (10.8%)	1.063 (1.012–1.117)	0.015	1.060 (1.008–1.114)	0.022
TVR	28 (10.0%)	1.061 (1.008–1.117)	0.023	1.056 (1.003–1.112)	0.037
TVMI	11 (3.9%)	0.993 (0.902–1.092)	0.878		
Stent thrombosis	4 (1.4%)	0.943 (0.788–1.127)	0.516		

HR, hazard ratio; TVF, target vessel failure; TVMI, target vessel myocardial infarction; TVR, target vessel revascularization; vFFR, vessel fractional flow reserve.

Values are hazard ratios (HRs) with 95% confidence intervals (CIs) per 0.01 decrease in residual vFFR.

⁎ Multivariable models were constructed only for outcomes with a statistically significant unadjusted association. TVF was adjusted for sex, hypertension, dyslipidemia, and diabetes; TVR or TVMI for sex, diabetes, and dyslipidemia; and TVR for sex and diabetes. For the remaining endpoints, adjustment was not performed because the number of events was too low to allow reliable multivariable modelling.

Sensitivity analyses using alternative residual vFFR thresholds (0.88, 0.90, and 0.92), parsimonious Cox models incorporating clinical history and lesion-level characteristics (Supplementary Table 6), and an LAD-only analysis (Supplementary Table 2) yielded findings consistent with the primary analysis.

At 5 years, in both unadjusted and adjusted models, patients with a post-PCI FFR <0.89 had a significantly higher incidence of TVF [25 (20.8%) vs. 15 (9.4%); HR 2.24, 95% CI 1.18–4.26; *p* = 0.013; adjusted HR 2.18, 95% CI 1.13–4.19; *p* = 0.020]. Cardiac death occurred in 9 (7.5%) vs. 5 (3.1%) patients (HR 2.36, 95% CI 0.79–7.04; *p* = 0.12). No statistically significant differences were observed in TVR or TVMI [18 (15.0%) vs. 12 (7.5%); HR 2.02, 95% CI 0.97–4.19; *p* = 0.06], TVR [17 (14.2%) vs. 11 (6.9%); HR 2.07, 95% CI 0.97–4.42; *p* = 0.06], TVMI [5 (4.2%) vs. 6 (3.8%); HR 0.88, 95% CI 0.34–3.60; *p* = 0.88], or ST [1 (0.8%) vs. 3 (1.9%); HR 0.43, 95% CI 0.05–4.17; *p* = 0.47]. The results of clinical outcomes for assessing the impact of post-PCI FFR are summarized in Supplementary Table 1 and Supplemetary Fig. 2.

## 4 Discussion

The FAST Residual study was conducted to investigate the impact of residual vFFR on clinical outcomes, and it is the first study to use virtual stenting with follow-up up to 5 years. The main findings can be summarized as follows: 1) Residual vFFR predicts adverse clinical outcomes at 5 years, 2) Patients with a residual vFFR <0.90 experienced significantly higher 5-year rates of TVF, TVR or TVMI, and TVR as compared to patients with a residual vFFR ≥0.90, and 3) The correlation between residual vFFR and post-PCI FFR was moderate.

The concept of angiography-based virtual stenting was first introduced by Gosling et al., who presented a simulated stent placement based on invasive coronary angiography views using the VIRTUheart workflow software, demonstrating a strong correlation between simulated FFR and post-PCI FFR (*r* = 0.80) [18]. The more recent AQVA study demonstrated that quantitative flow ratio (QFR)-based virtual PCI guidance was superior to angiography-guided PCI in achieving optimal post-PCI physiological outcomes, as defined by a post-PCI QFR value of <0.90 (6.6% vs. 15.1%; *p* = 0.009) [19]. Subsequent studies successfully employed simulated post-PCI FFR values derived from various angiography-based methods to predict long-term vessel-related adverse events [20], [21], [22]. The present study adds to these findings by presenting the first validation of the predictive value of residual vFFR on long-term clinical outcomes.

At first, we demonstrated a moderate correlation between residual vFFR and final post-PCI FFR (*r* = 0.51), which was lower than that reported in the initial validation study of vFFR virtual stenting [12]. However, correlations between simulated and measured post-PCI physiology have varied across angiography-based virtual PCI approaches. Reported correlations for QFR-based virtual PCI ranged from *r* = 0.16 in the QIMERA-I study to *r* = 0.53 in a substudy of the COE-PERSPECTIVE registry and *r* = 0.68 in the DOCTORS study, while simulated post-PCI angio-FFR demonstrated a correlation of *r* = 0.61 with measured post-PCI FFR [20], [21], [23], [24].

In line with prior reports, we observed a modest systematic difference between virtual and invasive measurements. The mean difference between residual vFFR and post-PCI FFR was **+0.015**, which is considerably smaller than that reported for QFR-based virtual PCI (**+0.068**) [21] and comparable in absolute magnitude to angio-FFR (**−0.010**), albeit in the opposite direction [20]. As residual vFFR is calculated following virtual stenting of the target lesion, a persistently low value may reflect pressure loss arising from disease in the untreated vessel segments and may therefore reflect residual vessel-level epicardial disease burden; however, it cannot by itself distinguish diffuse from focal disease. This interpretation should therefore be considered hypothesis-generating.The LAD and hydrostatic pressure effects and may reduce systematic differences between invasive and angiography-derived physiological indices [25], [26]. Another reason for this discrepancy between residual vFFR and post-PCI FFR can be explained by the assumption of optimal stenting conditions when calculating residual vFFR. The software assumes ideal stent deployment without geographic miss or underexpansion, yet intravascular imaging (IVUS) studies have shown that optimal stenting criteria are met in fewer than 50% of PCI cases [27], [28]. In routine practice, optimal stent implantation may be hampered by heavy calcifications, geographic miss and stent edge dissections [29], [30], [31]. In fact, severe calcification was present in 42% of patients in the present study, a condition more common in diffuse than focal coronary artery disease (CAD), which may contribute to the overestimation of residual vFFR [32], [33]. Previous studies have already demonstrated significantly lower post-PCI FFR values in cases of diffuse compared to focal CAD, as well as worse clinical outcomes [3], [4], [5], [6], [7], [8], [32], [34]. A low residual vFFR may reflect persistent pressure loss arising from diseased segments without focal lesions.

IVUS was used in 12.9% of patients in the present study, and plaque modification was mainly guided by angiography. The latter hampers our ability to verify the rationale for discrepancies between residual vFFR and post-PCI FFR. As such, the use of residual vFFR should rather inform the physician upfront on what could be reasonably achieved by optimal PCI. Conversely, the correlation between residual vFFR and post-PCI FFR should only be considered as hypothesis-generating and illustrates the need for intravascular imaging to increase the likelihood of optimal PCI results [35].

Despite known discrepancies between residual vFFR and post-PCI FFR, angiography-based virtual estimates of post-PCI FFR have consistently shown significant predictive value for assessing PCI outcomes. These findings remained consistent when the analyses were restricted to LAD vessels. Similarly, studies using simulated post-PCI FFR by methods like QFR or angioFFR have demonstrated its utility in predicting adverse clinical events [20], [21], [22]. A distinguishing feature of our study is the comparison of residual vFFR, derived from pre-PCI virtual stenting, with measured post-PCI FFR, while PCI itself was not guided by the virtual simulation. Although associations were numerically stronger for residual vFFR, this should not be interpreted as evidence of superior prognostic performance; for TVR, the effect estimates were similar, and the difference in statistical significance may partly reflect the limited number of events and different cut-off values. Rather, the two indices may provide complementary information, with residual vFFR estimating the physiological result potentially achievable after idealized PCI and reflecting residual vessel-level disease burden, whereas post-PCI FFR reflects the physiological result actually achieved after intervention. However, such comparisons must also take into account subtle differences in optimal cut-off values for defining PCI success or predicting outcomes. Our analysis revealed that the optimal threshold for post-PCI FFR (<0.89) was slightly lower than that for residual vFFR (<0.90), which is consistent with the lower median post-PCI FFR values (0.90) observed in our study compared to residual vFFR (0.92). These findings suggest a significant potential for solutions that allow the simulation of PCI outcomes in determining a final revascularization strategy, also stratifying patients with diffuse disease that would likely be better served with coronary artery bypass surgery. Future prospective evidence is needed to establish the additional value of virtual stenting versus conventional angiography or physiology-guided PCI.

## 5 Limitations

The present study was a post hoc analysis of the single-center observational FFR SEARCH study, resulting in several limitations: 1) obtaining adequate post-PCI angiographic views was not protocol-mandated, leading to the exclusion of 54.6% of patients. Although baseline clinical characteristics and 5-year vessel-related outcomes were largely comparable between included and excluded eligible patients, differences in selected angiographic and procedural characteristics were observed. Consequently, residual selection bias cannot be excluded, and the generalizability of our findings to an unselected PCI population may be limited. 2) Some baseline characteristics were not evenly distributed between the groups with a difference in the incidence of LAD lesions being most apparent. Although additional sensitivity analyses incorporating clinical history and lesion-level characteristics, as well as an LAD-only analysis, yielded findings consistent with the primary analysis, residual confounding cannot be excluded. 3.) The optimal residual vFFR cutoff was derived and evaluated within the same study population using the maximum log-rank statistic, which may result in optimistic estimation of its prognostic performance. Nevertheless, sensitivity analyses using alternative residual vFFR thresholds and modelling residual vFFR as a continuous variable yielded consistent associations with 5-year TVF, supporting the robustness of the observed prognostic relationship. Finally, residual vFFR assumes a perfect stent result, which is often unattainable due to factors such as heavy calcifications, geographic miss and stent edge dissections.

## 6 Conclusions

The concept of virtual stenting is a promising tool for predicting clinical outcomes. For the first time, this post hoc analysis showed that patients with residual vFFR below 0.90 experienced significantly higher rates of adverse clinical outcomes.

## CRediT authorship contribution statement

**Karol Sadowski:** Writing – review & editing, Writing – original draft, Visualization, Software, Methodology, Investigation, Formal analysis, Data curation, Conceptualization. **Jari A. van der Eijk:** Writing – review & editing, Writing – original draft, Software, Data curation. **Mariusz Tomaniak:** Writing – review & editing, Supervision, Resources, Funding acquisition. **Annemieke C. Ziedses des Plantes:** Writing – review & editing, Resources, Investigation, Data curation. **Wijnand K. den Dekker:** Writing – review & editing, Resources, Project administration, Investigation. **Roberto Diletti:** Writing – review & editing, Supervision, Project administration, Methodology, Formal analysis, Data curation, Conceptualization. **Rutger-Jan Nuis:** Writing – review & editing, Resources. **Jeroen Wilschut:** Writing – review & editing, Resources, Investigation. **Isabella Kardys:** Writing – review & editing, Supervision, Methodology, Formal analysis, Data curation. **Nicolas M. Van Mieghem:** Writing – review & editing, Supervision, Software, Resources, Project administration, Funding acquisition. **Joost Daemen:** Writing – review & editing, Supervision, Software, Resources, Project administration, Methodology, Funding acquisition, Conceptualization.

## Declaration of competing interest

The authors declare that they have no known competing financial interests or personal relationships that could have appeared to influence the work reported in this paper.

## Appendix A Supplementary data

Supplementary Figure 1. Optimal residual vFFR and post-PCI FFR cutoffs for predicting 5-year target vessel failure based on the maximum log-rank statistic.

Supplementary Figure 2. Kaplan-Meier curves for post-PCI FFR.

Supplementary Table 1. Clinical outcomes at 5-year follow-up according to post-PCI FFR.

Supplementary Table 2. Clinical outcomes at 5-year follow-up for residual vFFR in LAD vessels only.

Supplementary Table 3. Baseline clinical characteristics of the eligible study population stratified by inclusion in the final residual vFFR analysis.

Supplementary Table 4. Vessel-level angiographic and procedural characteristics according to feasibility of residual vFFR analysis.

Supplementary Table 5. Five-year clinical outcomes in patients included in and excluded from the final residual vFFR analysis.

Supplementary Table 6. Sensitivity analyses for the association between residual vFFR <0.90 and 5-year target vessel failure.
