# Supplementary material for: The impact of residual vessel fractional flow reserve on clinical outcomes: The FAST residual study
Source: Int J Cardiol Heart Vasc. 2026 Oct 6;67:102022. doi: 10.1016/j.ijcha.2026.102022 (PMC13676154; doi:10.1016/j.ijcha.2026.102022)
Supplement: Supplementary file 1 [file mmc1.docx]

## **Supplementary Figure 1. Optimal residual vFFR and post-PCI FFR cutoff to predict 5-year target vessel failure based on the maximum log-rank statistic.**

| **A** | **B** |
| --- | --- |

Supplementary Figure 1. Optimal residual vFFR (A) and post-PCI FFR (B) cutoff to predict 5-year target vessel failure based on the maximum log-rank statistic. FFR, fractional flow reserve; vFFR, vessel FFR.
