# Supplementary material for: The impact of residual vessel fractional flow reserve on clinical outcomes: The FAST residual study
Source: Int J Cardiol Heart Vasc. 2026 Oct 6;67:102022. doi: 10.1016/j.ijcha.2026.102022 (PMC13676154; doi:10.1016/j.ijcha.2026.102022)
Supplement: Supplementary file 2 [file mmc2.docx]

## **Supplementary Figure 2. Kaplan-Meier curves for post-PCI FFR**

| **A** | **B** |
| --- | --- |
| **C** | **D** |
| **E** | **F** |

Supplementary Figure 2. Kaplan-Meier curves based on post-PCI FFR for TVF (**A**), cardiac death (**B**), TVR or TVMI (**C**), TVR (**D**), TVMI (E) and stent thrombosis (F). The adjusted confounders were: for TVF – sex, hypertension, dyslipidemia, and diabetes. HR, hazard ratio; TVF, target vessel failure; TVMI, target vessel myocardial infarction; TVR, target vessel revascularization; res, residual; vFFR, vessel fractional flow reserve.
