# Supplementary material for: The impact of residual vessel fractional flow reserve on clinical outcomes: The FAST residual study
Source: Int J Cardiol Heart Vasc. 2026 Oct 6;67:102022. doi: 10.1016/j.ijcha.2026.102022 (PMC13676154; doi:10.1016/j.ijcha.2026.102022)
Supplement: Supplementary file 3 [file mmc3.docx]

## **Supplementary Table 1. Clinical Outcomes at 5-year Follow-Up for post-PCI FFR.**

|  | Post PCI $<$0.89 | Post PCI ≥0.89 | Univariate | | Multivariate | |
| --- | --- | --- | --- | --- | --- | --- |
|  | Patients with event n (%) | Patients with event n (%) | HR (95% CI) | P value | Adjusted HR* (95% CI) | p-value |
|  | N=120 (43.0%) | N=159 (57.0%) |  | | | |
| Cardiac death | 9 (7.5%) | 5 (3.1%) | 2.36 (0.79–7.04) | 0.12 |  |  |
| TVF | 25 (20.8%) | 15 (9.4%) | 2.24 (1.18–4.26) | 0.013 | 2.18 (1.13–4.19) | 0.020 |
| TVR or TVMI | 18 (15.0%) | 12 (7.5%) | 2.02 (0.97–4.19) | 0.06 |  |  |
| TVR | 17 (14.2%) | 11 (6.9%) | 2.07 (0.97–4.42) | 0.06 |  |  |
| TVMI | 5 (4.2%) | 6 (3.8%) | 0.88 (0.34–3.60) | 0.88 |  |  |
| Stent thrombosis | 1 (0.8%) | 3 (1.9%) | 0.43 (0.05–4.17) | 0.47 |  |  |
| HR, hazard ratio; TVMI, target vessel myocardial infarction; TVF, target vessel failure; TVR, target vessel revascularization.  * Multivariable models were constructed only for outcomes with a statistically significant unadjusted association. TVF was adjusted for sex, hypertension, dyslipidemia, and diabetes. | | | | | | |
