# Supplementary material for: The impact of residual vessel fractional flow reserve on clinical outcomes: The FAST residual study
Source: Int J Cardiol Heart Vasc. 2026 Oct 6;67:102022. doi: 10.1016/j.ijcha.2026.102022 (PMC13676154; doi:10.1016/j.ijcha.2026.102022)
Supplement: Supplementary file 4 [file mmc4.docx]

## **Supplementary Table 2. Clinical outcomes at 5-year follow-up for LAD vessels only for residual vFFR.**

|  | Residual vFFR $<$0.90 | Residual vFFR ≥0.90 | Univariate | | Multivariate | |
| --- | --- | --- | --- | --- | --- | --- |
|  | Patients with event n (%) | Patients with event n (%) | HR (95% CI) | p-value | Adjusted HR* (95% CI) | p-value |
|  | N=83 (48.3%) | N=88 (51.7%) |  | | | |
| Cardiac death | 7 (8.4%) | 2 (2.3%) | 3.84 (0.80–18.51) | 0.09 |  |  |
| TVF | 20 (24.1%) | 8 (9.1%) | 2.88 (1.27–6.55) | 0.011 | 2.74 (1.18–6.34) | 0.019 |
| TVR or TVMI | 14(16.9%) | 8 (9.1%) | 2.03 (0.85–4.84) | 0.11 |  |  |
| TVR | 13 (15.7%) | 7 (8.0%) | 2.14 (0.85–5.35) | 0.11 |  |  |
| TVMI | 4 (4.8%) | 3 (3.4%) | 1.47 (0.33–6.57) | 0.61 |  |  |
| Stent thrombosis | 1 (1.2%) | 0 (0%) | -** | -** |  |  |
| HR, hazard ratio; TVMI, target vessel myocardial infarction; TVF, target vessel failure; TVR, target vessel revascularization.  * Multivariable models were constructed only for outcomes with a statistically significant unadjusted association. TVF was adjusted for sex, hypertension, dyslipidemia, and diabetes.  **Stent thrombosis occurred in 1 patient. Due to the low number of events, hazard ratio estimates were not computed. | | | | | | |
