# Supplementary material for: The impact of residual vessel fractional flow reserve on clinical outcomes: The FAST residual study
Source: Int J Cardiol Heart Vasc. 2026 Oct 6;67:102022. doi: 10.1016/j.ijcha.2026.102022 (PMC13676154; doi:10.1016/j.ijcha.2026.102022)
Supplement: Supplementary file 5 [file mmc5.docx]

## Supplementary Table 3. Baseline clinical characteristics of the eligible study population stratified by inclusion in the final residual vFFR analysis.

|  | All eligible patients (n=614) | Included (n=279) | Excluded (n=335) | p-value |
| --- | --- | --- | --- | --- |
| Age, y | 64.95 ± 11.25 | 63.97 ± 10.79 | 65.76 ± 11.57 | 0.0496 |
| Male sex, n (%) | 428 (69.7%) | 191 (68.5%) | 237 (70.7%) | 0.54 |
| Cardiovascular risk factors, n (%) | | | | |
| Hypertension | 363 (59.4%) | 169 (60.8%) | 194 (58.3%) | 0.53 |
| Hypercholesterolemia | 331 (54.4%) | 153 (55.4%) | 178 (53.5%) | 0.63 |
| Diabetes | 147 (23.9%) | 66 (23.7%) | 81 (24.2%) | 0.88 |
| Current smoker | 273 (44.5%) | 131 (47.0%) | 142 (42.5%) | 0.27 |
| Prior stroke | 51 (8.3%) | 24 (8.6%) | 27 (8.1%) | 0.81 |
| Peripheral arterial disease | 52 (8.5%) | 23 (8.2%) | 29 (8.7%) | 0.86 |
| Comorbidity, n (%) | | | | |
| Prior myocardial infarction | 146 (23.8%) | 67 (24.0%) | 79 (23.6%) | 0.90 |
| Prior PCI | 193 (31.4%) | 83 (29.7%) | 110 (32.8%) | 0.41 |
| Prior CABG | 39 (6.4%) | 11 (3.9%) | 28 (8.4%) | 0.026 |
| Creatinine, µmol/L, median (IQR) | 84 [73–100] | 82 [72–98.25] | 86 [73–102] | 0.18 |
| Hb level, mmol/L, median (IQR) | 8.7 [8.0–9.3] | 8.8 [8.1–9.3] | 8.6 [8.0–9.3] | 0.19 |
| Presentation, n (%) | | | | |
| Stable angina | 278 (45.3%) | 122 (43.7%) | 156 (46.6%) | 0.48 |
| Unstable angina/NSTEMI | 336 (54.7%) | 157 (56.3%) | 179 (53.4%) | 0.48 |
| Data are reported as mean ± SD, median [IQR], or n (%), as appropriate.  CABG, coronary artery bypass grafting; Hb, haemoglobin; IQR, interquartile range; NSTEMI, non-ST-segment elevation myocardial infarction; PCI, percutaneous coronary intervention; SD, standard deviation. | | | | |
