# Supplementary material for: The impact of residual vessel fractional flow reserve on clinical outcomes: The FAST residual study
Source: Int J Cardiol Heart Vasc. 2026 Oct 6;67:102022. doi: 10.1016/j.ijcha.2026.102022 (PMC13676154; doi:10.1016/j.ijcha.2026.102022)
Supplement: Supplementary file 6 [file mmc6.docx]

## Supplementary Table 4. Vessel-level angiographic and procedural characteristics according to feasibility of residual vFFR analysis.

|  | **All vessels (n=761)** | **Analyzable (n=303)** | **Non-analyzable (n=458)** | **p-value** |
| --- | --- | --- | --- | --- |
| ACC/AHA lesion type, n (%) | | | | |
| A | 97 (12.7%) | 45 (14.9%) | 52 (11.4%) | 0.16 |
| B1 | 160 (21.0%) | 69 (22.8%) | 91 (19.9%) | 0.34 |
| B2/C | 504 (66.2%) | 189 (62.4%) | 315 (68.8%) | 0.07 |
| Bifurcation | 100 (13.1%) | 41 (13.5%) | 59 (12.9%) | 0.80 |
| Calcified | 316 (41.5%) | 125 (41.3%) | 191 (41.7%) | 0.90 |
| Thrombus | 74 (9.7%) | 23 (7.6%) | 51 (11.1%) | 0.11 |
| Measured vessel, n (%) | | | | |
| RCA | 194 (25.5%) | 67 (22.1%) | 127 (27.7%) | 0.08 |
| LAD | 373 (49.0%) | 174 (57.4%) | 199 (43.4%) | <0.001 |
| LCx | 180 (23.7%) | 58 (19.1%) | 122 (26.6%) | 0.017 |
| IM | 14 (1.8%) | 4 (1.3%) | 10 (2.2%) | 0.39 |
| QCA measurements; median (IQR) | | | | |
| Stenosis pre, %, mean±SD | 60.38 ± 19.56 | 55.21 ± 16.39 | 63.77 ± 20.72 | <0.001 |
| Stenosis post, %, mean±SD | 3.59 ± 13.73 | 3.70 ± 13.56 | 3.51 ± 13.86 | 0.86 |
| MLD pre, mm | 1.03 [0.71–1.39] | 1.11 [0.81–1.43] | 0.95 [0.60–1.35] | <0.001 |
| MLD post, mm | 2.55 [2.22–2.87] | 2.50 [2.18–2.81] | 2.59 [2.26–2.91] | 0.015 |
| Stent length, mm | 25 [15–38] | 22 [15–35] | 26 [18–40] | 0.014 |
| Stent diameter, mm | 3.00 [2.83–3.50] | 3.00 [2.75–3.50] | 3.00 [2.89–3.50] | 0.18 |
| No. of stents, n | 1 [1–2] | 1 [1–1] | 1 [1–2] | <0.001 |
| Predilatation, n (%) | 533 (70.0%) | 201 (66.3%) | 332 (72.5%) | 0.07 |
| Postdilatation, n (%) | 477 (62.8%) | 199 (65.9%) | 278 (60.7%) | 0.15 |
| Use of IVUS, n (%) | 80 (10.5%) | 36 (11.9%) | 44 (9.6%) | 0.31 |
| Post-PCI FFR | 0.90 [0.85–0.95] | 0.90 [0.85–0.95] | 0.91 [0.86–0.96] | 0.08 |
| Data are reported as mean ± SD, median [IQR], or n (%), as appropriate. FFR, fractional flow reserve; IM, intermediate artery; IQR, interquartile range; IVUS, intravascular ultrasound; LAD, left anterior descending artery; LCx, left circumflex artery; MLD, minimum luminal diameter; PCI, percutaneous coronary intervention; QCA, quantitative coronary angiography; RCA, right coronary artery; SD, standard deviation. | | | | |
