# Supplementary material for: The impact of residual vessel fractional flow reserve on clinical outcomes: The FAST residual study
Source: Int J Cardiol Heart Vasc. 2026 Oct 6;67:102022. doi: 10.1016/j.ijcha.2026.102022 (PMC13676154; doi:10.1016/j.ijcha.2026.102022)
Supplement: Supplementary file 7 [file mmc7.docx]

Supplementary Table 5. Five-year clinical outcomes in patients included and excluded from the final residual vFFR analysis.

|  | **Included (n=279)** | **Excluded (n=335)** | **Unadjusted HR (95% CI)** | **p-value** |
| --- | --- | --- | --- | --- |
| TVF | 41 (14.7%) | 51 (15.2%) | 0.93 (0.62-1.41) | 0.74 |
| Cardiac death | 14 (5.0%) | 29 (8.7%) | 0.55 (0.29-1.04) | 0.06 |
| TVR or TVMI | 31 (11.1%) | 28 (8.4%) | 1.29 (0.77-2.15) | 0.33 |
| TVR | 29 (10.4%) | 27 (8.1%) | 1.25 (0.74-2.11) | 0.40 |
| TVMI | 12 (4.3%) | 12 (3.6%) | 1.16 (0.52-2.58) | 0.72 |
| Stent thrombosis | 4 (1.4%) | 6 (1.8%) | 0.79 (0.22-2.79) | 0.71 |
| Data are reported as n (%). HRs with 95% CIs were estimated using univariable Cox proportional hazards regression, with excluded patients serving as the reference group. Follow-up was censored at the time of last contact or at 5 years, whichever occurred first. Vessel-related outcomes were assessed at the patient level as the occurrence of an event in any screened study vessel. TVF was defined as the composite of cardiac death, TVMI, and TVR. CI, confidence interval; HR, hazard ratio; ST, stent thrombosis; TVF, target vessel failure; TVMI, target vessel myocardial infarction; TVR, target vessel revascularization; vFFR, vessel fractional flow reserve. | | | | |
