# Supplementary material for: The impact of residual vessel fractional flow reserve on clinical outcomes: The FAST residual study
Source: Int J Cardiol Heart Vasc. 2026 Oct 6;67:102022. doi: 10.1016/j.ijcha.2026.102022 (PMC13676154; doi:10.1016/j.ijcha.2026.102022)
Supplement: Supplementary file 8 [file mmc8.docx]

Supplementary Table 6. Sensitivity analyses for the association between residual vFFR <0.90 and 5-year target vessel failure

| **Model** | **Covariates included in the model** | **Adjusted HR for residual vFFR <0.90** | **95% CI** | **p-value** |
| --- | --- | --- | --- | --- |
| Primary multivariable model | Sex, hypertension, dyslipidemia, diabetes | 2.66 | 1.41–5.04 | 0.003 |
| Sensitivity model 1 – clinical history | Prior MI, prior PCI | 2.45 | 1.29–4.66 | 0.006 |
| Sensitivity model 2 – lesion/procedural | LAD location, stent length | 2.82 | 1.41–5.63 | 0.003 |
| Sensitivity model 3 – lesion complexity | LAD location, ACC/AHA B2/C lesion complexity, stent length | 2.59 | 1.29–5.20 | 0.008 |
| Data are presented as adjusted HRs with 95% CIs. Sensitivity analyses were performed using Cox proportional hazards regression for the primary endpoint of 5-year TVF. Separate parsimonious models were constructed to account for differences in clinical history and lesion-level characteristics between residual vFFR groups. Residual vFFR ≥0.90 served as the reference category. Stent length was entered as a continuous variable.  ACC/AHA, American College of Cardiology/American Heart Association; CI, confidence interval; HR, hazard ratio; LAD, left anterior descending artery; MI, myocardial infarction; PCI, percutaneous coronary intervention; TVF, target vessel failure; vFFR, vessel fractional flow reserve. | | | | |
